# 48 or 370 to 1000 and More

**DOI:** 10.1063/4.0001080

**Published:** 2025-10-27

**Authors:** Lisa J. Keefe

**Affiliations:** AUI - Center for Advancing Therapeutics, Lemont, IL, USA

## Abstract

Structure is of vital importance to structure-based drug design, significantly impacting both target selection and the rate of drug development. Increasing numbers of drug development portfolios, growing numbers of pharmaceutical and biotech companies, and demands for faster development are key drivers for technology development in macromolecular crystallography. For more than 25 years, the Industrial Macromolecular Crystallography Association Collaborative Access Team (IMCA-CAT) has focused exclusively on accelerating drug discovery and development through synchrotron-based structural biology research. Ultra- high throughput is an important goal of the facility. In pursuing this goal, the leveraging of new technologies has driven further technology development, enabling expansion and pushing limits.

